# Editorial: Endothelial Dynamics in Health and Disease

**DOI:** 10.3389/fphys.2020.611117

**Published:** 2020-11-19

**Authors:** Elizabeth A. V. Jones, Mariona Graupera, Jaap D. van Buul, Stephan Huveneers

**Affiliations:** ^1^Centre for Molecular and Vascular Biology, KU Leuven, Leuven, Belgium; ^2^Department of Cardiology, Cardiovascular Research Institute Maastricht, Maastricht University, Maastricht, Netherlands; ^3^Vascular Biology and Signalling Group, ProCURE, Oncobell Program, Institut d'Investigació Biomèdica de Bellvitge (IDIBELL), L'Hospitalet de Llobregat, Barcelona, Spain; ^4^CIBERONC, Instituto de Salud Carlos III, Madrid, Spain; ^5^Sanquin Research and Landsteiner Laboratory, Leeuwenhoek Centre for Advanced Microscopy, Swammerdam Institute for Life Sciences, University of Amsterdam, Amsterdam, Netherlands; ^6^Department of Medical Biochemistry, Amsterdam Cardiovascular Sciences, Amsterdam University Medical Center, Location AMC, University of Amsterdam, Amsterdam, Netherlands

**Keywords:** endothelial cell, angiogenesis, VE-cadherin, cytoskeleton, mechanotransduction, VEGF signaling, lymphatic vessels, blood vessels

This special Research Topic of Frontiers in Physiology and Frontiers in Cell and Developmental Biology collates a series of review and research articles on the dynamic properties of endothelial cells, the key inner lining cells of blood and lymphatic vessels. Over the past decade, we have learnt that the vasculature is not a static tissue, but instead relies on the dynamic interactions of endothelial cells with each other, with the vascular microenvironment and with other cell types. For instance, new vessel formation occurs through collective migration and critically relies on rearrangements of interactions between endothelial cells. As such, endothelial cells are equipped with a variety of dynamic molecular systems that allow these cells to adapt to physiological and pathophysiological changes. Studying the systems that underlie endothelial dynamics brings fundamental insights into how vessels form and respond to their microenvironment. This knowledge is crucial to understand the development of cardiovascular disease, and it provides potential leads to guide the opening and closing of the vasculature for therapeutic applications in chronic inflammation and cancer.

Endothelial tissue integrity depends on adherens junctions that are based on the actin-anchored VE-cadherin receptor (Figure 1). The importance of the VE-cadherin complex has been widely studied in the context of endothelial barrier function (Orsenigo et al., 2012; Smith et al., 2020) (lymph)angiogenesis (Carmeliet et al., 1999; Bentley et al., 2014; Hägerling et al., 2018; Yang et al., 2019), inflammation (Wessel et al., 2014; van Steen et al., 2020), and flow sensing (Tzima et al., 2005; Lagendijk et al., 2017; Caolo et al., 2018). Duong and Vestweber now overview the complementary mechanisms that take place at endothelial cell-cell junctions beyond the VE-cadherin receptor in intact blood and lymphatic vessels. The importance of other adhesion receptors such as ESAM, Claudins and their anchoring to the dynamic actomyosin cytoskeleton is discussed as important contributors to endothelial integrity. In an original research paper by Werner et al., a new member of VE-cadherin-based endothelial junctions is identified: the actin-binding protein Coronin 1B. The authors investigated the molecular mechanism behind actomyosin relaxation and subsequent protrusive cytoskeletal activity evoked by Coronin 1B to seal the junctions.

**Figure 1:**
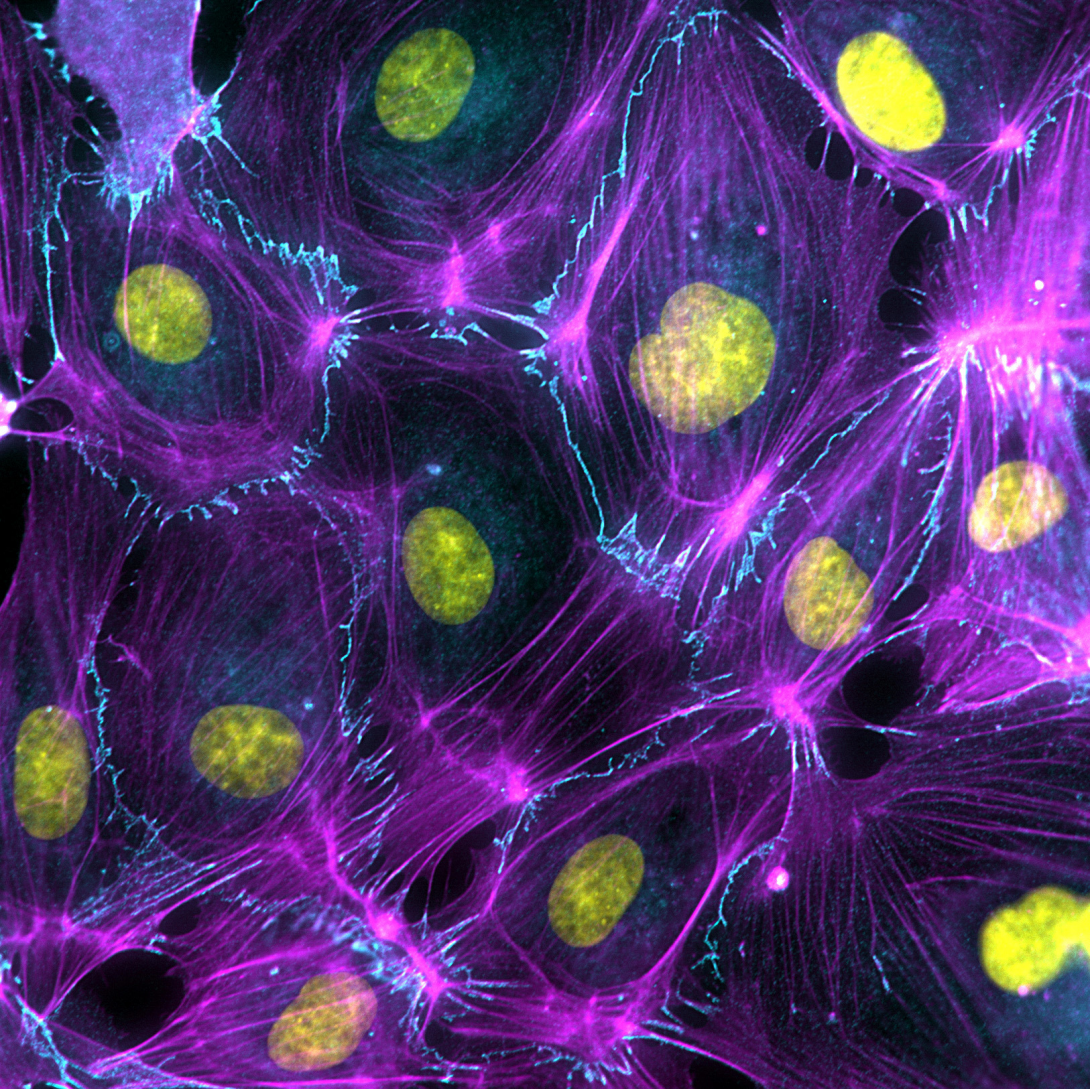
Endothelial cell dynamics is driven by the activity and organization of the actin cytoskeleton (Magenta), which anchors to VE-cadherin-based cell-cell junctions (Cyan).

Endothelial cell biology is controlled by VEGF signaling. Li et al. have investigated the importance of VEGF-A-mediated VEGFR2 activation and downstream VE-cadherin phosphorylation in experimental myocardial infarction models. Intriguingly, VEGFR2 Y949F knock-in mice are protected from cardiac edema, and these findings indicate that vascular leakage may be therapeutically targeted to improve heart tissue perfusion after infarction. The Neuropilins act as coreceptors for the VEGFRs and are well-known to support angiogenic signaling (Lampropoulou and Ruhrberg, 2014; Simons et al., 2016). Alghamdi et al. have investigated the angiogenic function of NRP2. The authors show that NRP2 promotes endothelial cell migration through regulating the trafficking of the fibronectin-binding integrin α5β1.

Integrins mediate the connection between the vascular extracellular matrix (ECM) and the contractile actomyosin cytoskeleton. Tight crosstalk occurs between endothelial adhesion structures during mechanotransduction: integrins translate forces from the ECM to actomyosin-based contractility, which in turn controls pulling forces on endothelial cell-cell contacts (Huveneers et al., 2015). Failure to respond to forces has direct consequences for vessel development and barrier function and underlies stiffness-related cardiovascular disease. Gordon et al. discuss the forces at play in vascular biology and provide a detailed overview of currently available advanced tools to study force-dependent events in blood and lymphatic endothelial cells cultured *in vitro* under physiological relevant conditions.

To be able to fully understand the impact of blood flow-derived forces on vascular development, one has to turn to *in vivo* experimental models. The review by Campinho et al. elaborates on the importance of various flow-derived mechanical cues on endothelial cell polarization, migration, cell shape changes, and proliferative functions at a single cell level. Endothelial responses to flow are outlined within the context of sprouting angiogenesis, intussusceptive angiogenesis, anastomosis, lumen formation, vessel stabilization, vessel size, and endothelial to haematopoietic transitions. An original research paper by Lv et al. addresses the correlations between flow-mediated dilation, polymorphisms in the gene encoding angiotensin-converting enzyme and endothelial secreted angiotensin II levels in pre-menopausal women.

It is increasingly clear that live imaging is necessary to understand the temporal endothelial activities in the developing vasculature and to this end zebrafish have become a powerful model system. Okuda and Hogan focus on new tools and insights in endothelial dynamics in vascular cell biology based on studies in zebrafish. A detailed toolbox of available genetic models to study endothelial properties during various stages of angiogenesis and their role in vascular development and disease are provided. Live imaging in zebrafish have also revealed exciting new concepts in our understanding of the origin of lymphatic vessels in tumors. In the review from Gutierrez-Miranda and Yaniv, the various endothelial and non-endothelial origins of lymphatics and their contribution to physiological development of the lymphatics and cancer lymphangiogenesis are discussed.

Genetic interference and fluorescence-based endothelial monitoring has led to impressive insights of endothelial cell dynamics during angiogenesis and vascular repair (Eilken and Adams, 2010; Simons et al., 2015; Angulo-Urarte et al., 2018). In the final review of this Research Topic Garcia-Gonzalez et al. compared the pro's and con's of the currently available state-of-the-art conditional genetic mouse models to investigate vascular dynamics at single cell and high molecular resolution. A historical perspective is provided as well as a comprehensive overview of the current sophisticated reporter tools to fluorescently label individual cells and perform lineage tracings in the vasculature (e.g., endothelial cells, pericytes, cardiomyocytes, or vascular smooth muscle cells) in combination with specific loss- or gain-of-function of molecular players.

Taken together, the field is poised to address many key outstanding questions in endothelial and vascular biology. Many previous studies have focused on the dynamics of endothelial cells during the initiation of sprouting in newly forming vasculature. But what happens at intermediate stages of vessel formation is less well-studied. Are comparable molecular systems responsible for vascular elongation and maintenance? Are there differences in endothelial dynamics between blood and lymphatic vessels? Which steps are decided on single cell level, and which require collective responses? We anticipate that current mechanistic insights and endothelial models allow the next generation of vascular researchers to address these caveats in the field of vascular biology and disease.

## Author Contributions

This manuscript was written by all authors. All authors have performed editorial reviewing tasks for this Research Topic.

## Conflict of Interest

MG is a consultant for Medicxi (London, UK) and has a research agreement with Merck & Co. Inc (North Wales, PA, USA) and with Venthera (Palo Alto, CA, USA). The remaining authors declare that the research was conducted in the absence of any commercial or financial relationships that could be construed as a potential conflict of interest.
